# Supplementary material for: Extinction debts and colonization credits of non-forest plants in the European Alps
Source: Nat Commun. 2019 Sep 20;10:4293. doi: 10.1038/s41467-019-12343-x (PMC6754411; doi:10.1038/s41467-019-12343-x)
Supplement: Supplementary file 1 — Supplementary Information [file 41467_2019_12343_MOESM1_ESM.pdf]

Supplementary Information

Extinction debts and colonization credits of non-forest plants in the European Alps

Rumpf *et al.*

Supplementary Table 1 Relations between disequilibria and species-specific properties of 135 vascular Alpine plants species. Extinction debts and colonization credits are relative to expected extinction and colonization events, respectively. Estimates were derived from beta-distributed linear regressions with a logit link for which values of extinction debts and colonization credits were transformed following Smithson and Verkuilen[1] to include zeros (see Methods). For dispersal capability high values represent higher dispersal capability of species by wind and animals, and for persistence capability high values represent competitive, long-living and dominant species. Temperature indicators represent alpine to nival (1), lower alpine to upper subalpine (1.5), subalpine (2), lower subalpine to upper montane (2.5), and montane (3) species. Values for dispersal and persistence capability were derived from own measurements, literature[2, 3, 4, 5, 6, 7, 8, 9, 10, 11, 12, 13, 14, 15] and online data bases[16, 17, 18, 19, 20, 21, 22, 23, 24, 25, 26, 27, 28, 29, 30, 31]. Temperature indicator values were derived from Landolt *et al.*[8].

Type of disequilibrium	Predictor	Estimate ± SE	df	z-value	p-value	pseudo R^2
Colonization credit	Intercept	-1.36 ± 0.13				
	Dispersal capability	-0.13 ± 0.06	131	-2.10	0.036	0.07
Extinction debt	Intercept	-2.16 ± 0.11				
	Dispersal capability	0.10 ± 0.05	131	1.92	0.055	0.03
Colonization credit	Intercept	-1.35 ± 0.13				
	Persistence capability	0.03 ± 0.09	131	0.33	0.741	<0.01
Extinction debt	Intercept	-2.16 ± 0.11				
	Persistence capability	-0.10 ± 0.07	131	-1.31	0.192	0.02
Colonization credit	Intercept	4.89 ± 1.05				
	Historical optimum	-2.84e-3 ± 0.48e-3	132	-5.94	<0.001	0.36
Extinction debt	Intercept	-6.87 ± 0.90				
	Historical optimum	2.10e-3 ± 0.40e-3	132	5.31	<0.001	0.20
Colonization credit	Intercept	-3.06 ± 0.35				
	Temperature indicator	0.98 ± 0.19	127	5.21	<0.001	0.28
Extinction debt	Intercept	-1.09 ± 0.27				
	Temperature indicator	-0.65 ± 0.16	127	-4.17	<0.001	0.14

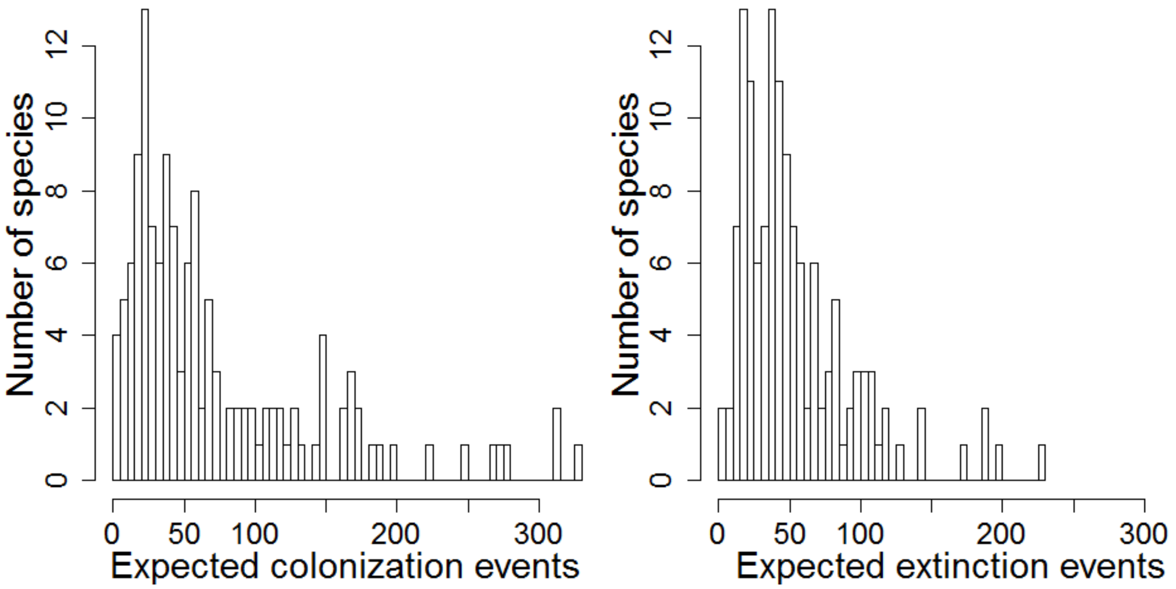

Supplementary Figure 1 Number of re-surveyed plots in which colonization (left) and extinction (right) events were expected for 135 vascular plant species of the European Alps. On average across all species, colonization events were expected in 73 of the 1576 re-surveyed plots and extinction events in 54. Nine and four species had an expected colonization and extinction event in less than 10 plots, respectively (colonization: *Carex rupestris*, *Gentiana orbicularis*, *Gentianella aspera*, *Leontodon montanus*, *Pedicularis aspleniifolia*, *Phyteuma globulariifolium*, *Primula integrifolia*, *Saxifraga moschata*, and *Sesleria ovata*; extinction: *Laserpitium halleri*, *Potentilla grandiflora*, *Rumex scutatus*, and *Valeriana celtica*), and no species had none expected colonization or extinction event.

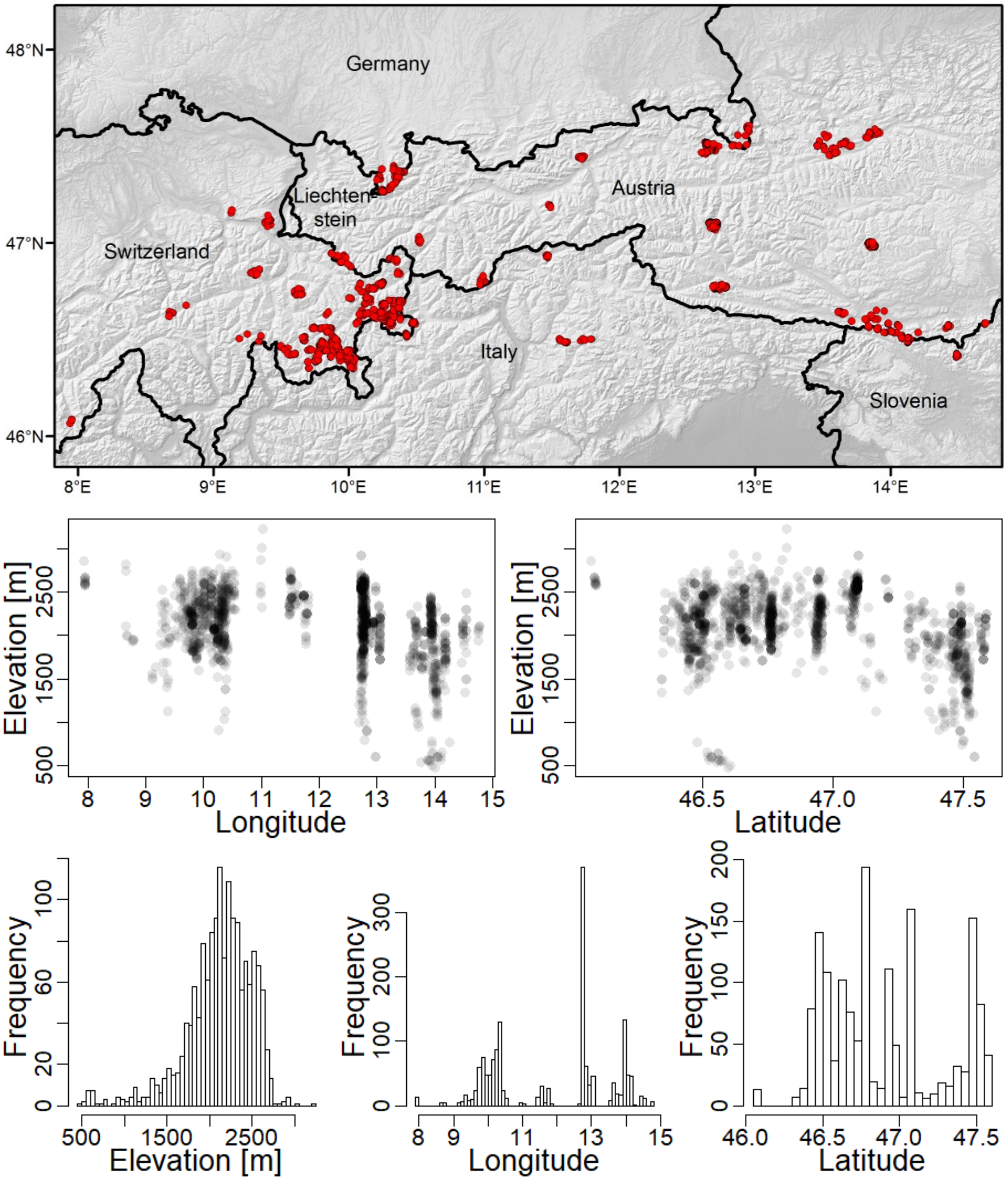

Supplementary Figure 2 Spatial distribution of the 1576 re-surveyed plots. Historical species distribution data were obtained from 26 historical publications[32, 33, 34, 35, 36, 37, 38, 39, 40, 41, 42, 43, 44, 45, 46, 47, 48, 49, 50, 51, 52, 53, 54, 55, 56, 57], were first recorded between 1911-1970, and none of these plots was re-surveyed multiple times. Below the tree line, plots were typically located on extensively used pastures during both the historical and recent survey. In the historical survey, the number of species per plot ranged from 1 to 70 with a mean of 24 species, while

the number of species per plot ranged from 2 to 81 with a mean of 29 in the recent re-survey. Potential sources of sampling bias and observer error were reduced as far as possible by a rigid re-location and re-survey design (see Rumpf *et al.*[58] for further details). The topographic background of the map was derived from Copernicus data of the European Environmental Agency.

Supplementary Methods

SDM calibration

```
BIOMOD_Modeling(myBiomodData,
                models              = c("GLM", "GAM", "GBM","CTA", "ANN",
                                        "SRE", "FDA", "MARS", "RF"),
                models.options      = *myBiomodOption*,
                NbRunEval           = 3,
                DataSplit           = 80,
                Prevalence          = 0.5,
                VarImport           = 3,
                models.eval.meth    = c("TSS", "ROC", "KAPPA"),
                SaveObj             = F,
                rescal.all.models   = T,
                do.full.models      = F)
```

where **myBiomodOption** are the default settings as follows:

```
GLM =   list( type = 'quadratic',
        interaction.level = 0,
        myFormula = NULL,
        test = 'AIC',
        family = binomial(link = 'logit'),
        mustart = 0.5,
        control = glm.control(epsilon = 1e-08, maxit = 50, trace = FALSE) ),
GAM =   list( algo = 'GAM_mgcv',
        type = 's_smoother',
        k = -1,
        interaction.level = 0,
        myFormula = NULL,
        family = binomial(link = 'logit'),
        method = 'GCV.Cp',
        optimizer = c('outer','newton'),
        select = FALSE,
        knots = NULL,
        paraPen = NULL,
        control = list(nthreads = 1, irls.reg = 0, epsilon = 1e-07, maxit = 200, trace = FALSE, mgc
                v.tol = 1e-07, mgcv.half = 15, rank.tol = 1.49011611938477e-08, nlm = list(ndi
                git=7, gradtol=1e-06, stepmax=2, steptol=1e-04, iterlim=200, check.analytical
                s=0), optim = list(factr=1e+07), newton = list(conv.tol=1e-06, maxNstep=5, m
                axSstep=2, maxHalf=30, use.svd=0), outerPIsteps = 0, idLinksBases = TRUE,
                scalePenalty = TRUE, keepData = FALSE, scale.est = pearson) ),
GBM =   list( distribution = 'bernoulli',
        n.trees = 2500,
        interaction.depth = 7,
        n.minobsinnode = 5,
        shrinkage = 0.001,
        bag.fraction = 0.5,
        train.fraction = 1,
        cv.folds = 3,
        keep.data = FALSE,
        verbose = FALSE,
        perf.method = 'cv'),
```

```
CTA =     list( method = 'class',
          parms = 'default',
          cost = NULL,
          control = list(xval = 5, minbucket = 5, minsplit = 5, cp = 0.001, maxdepth = 25) ),
ANN =     list( NbCV = 5,
          size = NULL,
          decay = NULL,
          rang = 0.1,
          maxit = 200),
SRE =     list( quant = 0.025),
FDA =     list( method = 'mars'),
MARS =    list( degree = 2,
          nk = NULL,
          penalty = 2,
          thresh = 0.001,
          prune = TRUE),
RF =      list( do.classif = TRUE,
          ntree = 500,
          mtry = 'default',
          nodesize = 5,
          maxnodes = NULL)
```

Ensemble modelling

```
BIOMOD_EnsembleModeling(modeling.output                 = myBiomodModelOut,
                        chosen.models                   = "all",
                        em.by                           = "all",
                        eval.metric                     = c("TSS"),
                        eval.metric.quality.threshold   = c(0.6),
                        prob.mean                       = T,
                        prob.cv                         = T,
                        prob.ci                         = F,
                        prob.ci.alpha                   = 0.05,
                        prob.median                     = F,
                        committee.averaging             = F,
                        prob.mean.weight                = T,
                        prob.mean.weight.decay          = "proportional")
```

Projections

```
BIOMOD_Projection(modeling.output       = myBiomodModelOut,
                  new.env               = *environmental_data*,
                  proj.name             = *running_name*,
                  selected.models       = "all",
                  binary.meth           = "TSS",
                  compress              = "xz",
                  keep.in.memory        = FALSE,
                  on_0_1000             = TRUE,
                  build.clamping.mask   = T)
```

where **environmental_data** refers to either historic or recent environmental data of the data set, and **running_name** to an arbitrary name to save the output.

Supplementary References

1. Smithson M, Verkuilen J. A better lemon squeezer? Maximum-likelihood regression with beta-distributed dependent variables. *Psychological Methods* **11**, 54-71 (2006).

2. Bruun HH, Österdahl S, Moen J, Angerbjörn A. Distinct patterns in alpine vegetation around dens of the Arctic fox. *Ecography* **28**, 81-87 (2005).

3. Cerabolini B, Ceriani RM, Caccianiga M, Andreis RD, Raimondi B. Seed size, shape and persistence in soil: a test on Italian flora from Alps to Mediterranean coasts. *Seed Science Research* **13**, 75-85 (2003).

4. Erschbamer B, Winkler J, Wagner J. Vegetative und generative Entwicklung von drei *Carex curvula*-Sippen in den Zentralalpen. *Flora* **189**, 277-286 (1994).

5. Hegi G. *Illustrierte Flora von Mitteleuropa*. Weissdorn-Verlag (1966-2008).

6. Hintze C, Heydel F, Hoppe C, Cunze S, König A, Tackenberg O. D3: The Dispersal and Diaspore Database - Baseline data and statistics on seed dispersal. *Perspectives in Plant Ecology Evolution and Systematics* **15**, 180-192 (2013).

7. Kutschera L, Lichtenegger E. *Wurzelatlas mitteleuropäischer Grünlandpflanzen*. Gustav Fischer Verlag (1982).

8. Landolt E*, et al. Flora indicativa - Ecological indicator values and biological attributes of the Flora of Switzerland and the Alps*, 2 edn. Haupt-Verlag (2010).

9. Luftensteiner HW. Untersuchungen zur Verbreitungsbiologie von Pflanzengemeinschaften an vier Standorten in Niederösterreich. *Bibliotheca Botanica* **135**, 1-68 (1982).

10. Müller-Schneider P. Verbreitungsbiologie der Blütenpflanzen Graubündens. *Veröffentlichungen des Geobotanischen Instituts der ETH, Stiftung Rübel* **85**, 1:263 (1986).

11. Pluess AR, Schütz W, Stöcklin J. Seed Weight Increases with Altitude in the Swiss Alps between Related Species but Not among Populations of Individual Species. *Oecologia* **144**, 55-61 (2005).

12. Römermann C, Tackenberg O, Poschlod P. How to predict attachment potential of seeds to sheep and cattle coat from simple morphological seed traits. *Oikos* **110**, 219-230 (2005).

13. Schroeter C. *Das Pflanzenleben der Alpen*. Albert Raustein (1926).

14. Tackenberg O. *Methoden zur Bewertung gradueller Unterschiede des Ausbreitungspotentials von Pflanzenarten: Modellierung des Windausbreitungspotentials und regelbasierte Ableitung des Fernausbreitungspotentials*. J. Cramer (2001).

15. Tamme R*, et al.* Predicting species' maximum dispersal distances from simple plant traits. *Ecology* **95**, 505-513 (2014).

16. Bjorkman AD*, et al.* Tundra Trait Team: A database of plant traits spanning the tundra biome. *Global Ecology and Biogeography* **27**, 1402-1411 (2018).

17. Cornelissen JHC, Diez PC, Hunt R. Seedling growth, allocation and leaf attributes in a wide range of woody plant species and types. *Journal of Ecology* **84**, 755-765 (1996).

18. Cornelissen JHC*, et al.* Functional traits of woody plants: correspondence of species rankings between field adults and laboratory-grown seedlings? *Journal of Vegetation Science* **14**, 311-322 (2003).

19. Diaz S*, et al.* The plant traits that drive ecosystems: Evidence from three continents. *Journal of Vegetation Science* **15**, 295-304 (2004).

20. Fitter AH, Peat HJ. The Ecological Flora Database. *Journal of Ecology* **82**, 415-425 (1994).

21. Garnier E*, et al.* Assessing the effects of land-use change on plant traits, communities and ecosystem functioning in grasslands: A standardized methodology and lessons from an application to 11 European sites. *Annals of Botany* **99**, 967-985 (2007).

22. Green W. USDA PLANTS Compilation. In: *NRCS: The PLANTS Database*. 09-02-02 edn. National Plant Data Center: Baton Rouge, USA (2009).

23. Kattge J*, et al.* TRY – a global database of plant traits. *Global Change Biology* **17**, 2905-2935 (2011).

24. Kleyer M*, et al.* The LEDA Traitbase: a database of life-history traits of the Northwest European flora. *Journal of Ecology* **96**, 1266-1274 (2008).

25. Klimešová J, de Bello F. CLO-PLA: the database of clonal and bud bank traits of Central European flora. *Journal of Vegetation Science* **20**, 511-516 (2009).

26. Kühn I, Durka W, Klotz S. BiolFlor - a new plant-trait database as a tool for plant invasion ecology. *Diversity and Distributions* **10**, 363-365 (2004).

27. Medlyn BE*, et al.* Stomatal conductance of forest species after long-term exposure to elevated CO2 concentration: A synthesis. *New Phytologist* **149**, 247-264 (2001).

28. Moretti M, Legg C. Combining plant and animal traits to assess community functional responses to disturbance. *Ecography* **32**, 299-309 (2009).

29. Royal Botanical Gardens KEW. Seed Information Database (SID). Royal Botanical Gardens KEW (2008).

30. Schweingruber FH, Landolt W. The Xylem Database. Swiss Federal Research Institute WSL (2005).

31. Wirth C, Lichstein JW. The Imprint of Species Turnover on Old-Growth Forest Carbon Balances - Insights From a Trait-Based Model of Forest Dynamics. In: *Old-Growth Forests: Function, Fate and Value* (ed^(eds Wirth C, Gleixner G, Heimann M). Springer Berlin Heidelberg (2009).

32. Lippert W. Die Pflanzengesellschaften des Naturschutzgebietes Berchtesgaden. *Berichte der Bayrischen Botanischen Gesellschaft* **39**, 67-122 (1966).

33. Albrecht J. *Soziologische und ökologische Untersuchungen alpiner Rasengesellschaften insbesondere auf Kalk-Silikat-Gesteinen*. Cramer (1969).

34. Zollitsch B. Soziologische und ökologische Untersuchungen auf Kalkschiefern in hochalpinen Gebieten. Teil I. *Berichte der Bayerischen Botanischen Gesellschaft* **40**, 67-100 (1968).

35. Pallmann H, Haffter P. Pflanzensoziologische und bodenkundliche Untersuchungen im Oberengadin: mit besonderer Berücksichtigung der Zwergstrauchgesellschaften der Ordnung Rhodoreto-Vaccinietalia. *Berichte der Schweizerischen Botanischen Gesellschaft* **42**, 357-466 (1933).

36. Schnyder A. Floristische und Vegetationsstudien im Alviergebiet. *Vierteljahresschrift der Naturforschenden Gesellschaft in Zürich* **17**, Beiblatt (1930).

37. Wagner H. Pflanzensoziologische Beobachtungen in der Ramsau bei Schladming. *Rundbrief der Zentralstelle für Vegetationskartierung des Reiches* **14**, Beilage (1944).

38. Wagner H. Die Pflanzendecke der Komperdellalm in Tirol. *Documents pour la carte de la végétation des Alpes* **3**, 7-59 (1965).

39. Lüdi W. *Die Pflanzengesellschaften der Schinigeplatte bei Interlaken und ihre Beziehungen zur Umwelt: eine vergleichend ökologische Untersuchung*. Geobotanisches Forschungsinstitut Rübel (1948).

40. Flütsch P. Über die Pflanzengesellschaften der alpinen Stufe des Berninagebietes. *Jahresbericht der Naturforschenden Gesellschaft Graubündens* **68**, 5-59 (1930).

41. Wikus E. Die Pflanzengesellschaften der Lienzer Dolomiten oberhalb der Baumgrenze. University of Vienna (1952).

42. Wendelberger G. Über einige hochalpine Pioniergesellschaften aus der Glockner- und Muntanitzgruppe in den Hohen Tauern. *Verhandlungen der Zoologisch-Botanischen Gesellschaft Österreichs* **93**, 100-109 (1953).

43. Thimm I. Die Vegetation des Sonnwendgebirges (Rofan) in Tirol (subalpine und alpine Stufe). *Berichte des Naturwissenschaftlich-Medizinischen Vereins Innsbruck* **50**, 5-166 (1953).

44. Pignatti-Wikus E. Pflanzensoziologische Studien im Dachsteingebiet. *Beiträge zur alpinen Karstforschung* **13**, 87-168 (1960).

45. Oberdorfer E. Borstgras- und Krummseggenrasen in den Alpen. *Beiträge zur naturkundlichen Forschung in Südwestdeutschland* **18**, 117-143 (1959).

46. Oberdorfer E. Beitrag zur Vegetationskunde des Allgäu. *Beiträge zur naturkundlichen Forschung in Südwestdeutschland* **9**, 29-98 (1950).

47. Höpflinger F. Die Pflanzengesellschaften des Grimminggebietes. *Mitteilungen des Naturwissenschaftlichen Vereins für Steiermark* **87**, 74-113 (1957).

48. Hartl H. Die Vegetation des Eisenhuts im Kärntner Nockgebiet. *Carinthia II* **73**, 293-336 (1963).

49. Gumpelmayer F. Die Vegetation und ihre Gliederung in den Leoganger Steinbergen. Universität Innsbruck (1967).

50. Braun-Blanquet J, Pallmann H, Bach R. *Pflanzensoziologische und bodenkundliche Untersuchungen im Schweizerischen Nationalpark und seinen Nachbargebieten: Vegetation und Böden der Wald- und Zwergstrauchgesellschaften (Vaccinio-Piceetalia)*. Nationalpark-Museum (1954).

51. Braun-Blanquet J, Jenny J. Vegetations-Entwicklung und Bodenbildung in der alpinen Stufe der Zentralalpen (Klimaxgebiet des Caricion curvulae): mit besonderer Berücksichtigung der Verhältnisse im schweizerischen Nationalparkgebiet. *Denkschriften der Schweizerischen Naturforschenden Gesellschaft* **63**, 183-349 (1926).

52. Braun-Blanquet J, Braun-Blanquet G, Trepp W, Bach R, F. R. Pflanzensoziologische und bodenkundliche Beobachtungen im Samnaun. *Jahresbericht der Naturforschenden Gesellschaft Graubündens* **90**, 3-48 (1964).

53. Braun-Blanquet J. *Die Pflanzengesellschaften der rätischen Alpen im Rahmen ihrer Gesamtverbreitung. Teil I.* Bischofberger (1969).

54. Braun-Blanquet G, Braun-Blanquet J. Recherches phytogéographiques sur le massif du Gross Glockner (Hohe Tauern). *Revue de géographie alpine* **19**, 675-735 (1931).

55. Braun J. Die Vegetationsverhältnisse der Schneestufe in den Rätisch-Lepontischen Alpen : Ein Bild des Pflanzenlebens an seinen äussersten Grenzen. *Neue Denkschrift der Schweizerischen Naturforschenden Gesellschaft* **48**, 1-339 (1913).

56. Aichinger E. *Vegetationskunde der Karawanken*. Verlag von Gustav Fischer (1933).

57. Aichinger E. Pflanzensoziologische Studien am Südfuß der Hochalmspitze. *Carinthia* **II**, 120-139 (1958).

58. Rumpf SB*, et al.* Range dynamics of mountain plants decrease with elevation. *Proceedings of the National Academy of Sciences* **115**, 1848-1853 (2018).
