# Supplementary material for: Extinction debts and colonization credits of non-forest plants in the European Alps
Source: Nat Commun. 2019 Sep 20;10:4293. doi: 10.1038/s41467-019-12343-x (PMC6754411; doi:10.1038/s41467-019-12343-x)
Supplement: Supplementary file 3 — Description of Additional Supplementary Files [file 41467_2019_12343_MOESM3_ESM.pdf]

Description of Additional Supplementary Files

File Name: Supplementary Data 1
Description: **Climatic data and values of calcareousness used for species distribution modelling**.
Note that re-survey data and additional plot-related data are available online in the Phaidra database at https://phaidra.univie.ac.at/view/o:630655. The columns 'RELEVE_NR', 'LONGITUDE', and 'LATITUDE' match those of the re-survey data. The remaining columns refer to the four variables minimum temperature of the coldest month (BioClim6; $°C^{-1}$), mean temperature of the warmest quarter (BioClim10; $°C^{-1}$), precipitation of the warmest quarter (BioClim18; mm), and calcareousness (%). Climatic variables are averages of the ten years preceding the historical survey ('Historical') or recent re-survey ('Recent'), and calcareousness was assumed to be constant over time. See Methods for further details.

File Name: Supplementary Data 2
Description: **Predictive accuracy of ensemble models parameterized with historical species distributions and environmental data.**
Given are mean True Skill Statistic scores[3] (TSS) and the number of successful models per species. An ensemble model was classified as successful when at least one of the contributing individual models reached a TSS > 0.6. The maximum number of successful models is 100 (see Methods for further details). Comparing model projections and current species observations resulted in a mean sensitivity of 0.45, a mean specificity of 0.88 and a mean TSS of 0.33 across all 135 species. Nomenclature follows Fischer *et al.*[1], supplemented by Lauber *et al.*[2].

References

1 Fischer, M. A., Oswald, K. & Adler, W. *Exkursionsflora für Österreich, Liechtenstein und Südtirol*. 3 edn, (Biologiezentrum der oberösterreichischen Landesmuseen, 2008).

2 Lauber, K., Wagner, G. & Gygax, A. *Flora Helvetica*. 5 edn, (Haupt Verlag, 2012).

3 Allouche, O., Tsoar, A. & Kadmon, R. Assessing the accuracy of species distribution models: prevalence, kappa and the true skill statistic (TSS). *Journal of Applied Ecology* **43**, 1223-1232 (2006).
