# Supplementary material for: Extinction debts and colonization credits of non-forest plants in the European Alps
Source: Nat Commun. 2019 Sep 20;10:4293. doi: 10.1038/s41467-019-12343-x (PMC6754411; doi:10.1038/s41467-019-12343-x)
Supplement: Supplementary file 6 — Reporting Summary [file 41467_2019_12343_MOESM6_ESM.pdf]

nature research

Corresponding author(s): Sabine Rumpf

Last updated by author(s): Aug 20, 2019

Reporting Summary

Nature Research wishes to improve the reproducibility of the work that we publish. This form provides structure for consistency and transparency in reporting. For further information on Nature Research policies, see Authors & Referees and the Editorial Policy Checklist.

Statistics

For all statistical analyses, confirm that the following items are present in the figure legend, table legend, main text, or Methods section.

n/a	Confirmed	
☐	☒	The exact sample size (*n*) for each experimental group/condition, given as a discrete number and unit of measurement
☐	☒	A statement on whether measurements were taken from distinct samples or whether the same sample was measured repeatedly
☐	☒	The statistical test(s) used AND whether they are one- or two-sided<br>*Only common tests should be described solely by name; describe more complex techniques in the Methods section.*
☐	☒	A description of all covariates tested
☐	☒	A description of any assumptions or corrections, such as tests of normality and adjustment for multiple comparisons
☐	☒	A full description of the statistical parameters including central tendency (e.g. means) or other basic estimates (e.g. regression coefficient) AND variation (e.g. standard deviation) or associated estimates of uncertainty (e.g. confidence intervals)
☐	☒	For null hypothesis testing, the test statistic (e.g. F, t, r) with confidence intervals, effect sizes, degrees of freedom and P value noted<br>*Give P values as exact values whenever suitable.*
☒	☐	For Bayesian analysis, information on the choice of priors and Markov chain Monte Carlo settings
☒	☐	For hierarchical and complex designs, identification of the appropriate level for tests and full reporting of outcomes
☐	☒	Estimates of effect sizes (e.g. Cohen's *d*, Pearson's *r*), indicating how they were calculated

Our web collection on statistics for biologists contains articles on many of the points above.

Software and code

Policy information about availability of computer code

Data collection	Re-survey coordintaes were determined using ArcGIS, version 10.2.2.3552.
Data analysis	All analyses were conducted in the programming environment R version 3.1.3. The following packages were used: biomod2, ade4, missMDA, betareg. See Methods for further details.

For manuscripts utilizing custom algorithms or software that are central to the research but not yet described in published literature, software must be made available to editors/reviewers. We strongly encourage code deposition in a community repository (e.g. GitHub). See the Nature Research guidelines for submitting code & software for further information.

Data

Policy information about availability of data

All manuscripts must include a data availability statement. This statement should provide the following information, where applicable:

- Accession codes, unique identifiers, or web links for publicly available datasets
- A list of figures that have associated raw data
- A description of any restrictions on data availability

Re-survey data are available online in the Phaidra database at https://phaidra.univie.ac.at/view/o:630655. The source data underlying Figures 1-3 are provided as a Source Data file. Climatic data and values of calcareousness used for species distribution modelling are provided in Supplementary Data 1. Trait values used to calculate species' dispersal and persistence capability are partly derived from the online database TRY. Following the intellectual property guidelines of TRY, raw trait data are not publicly available but can be obtained from the authors upon request and consent from TRY.

Field-specific reporting

Please select the one below that is the best fit for your research. If you are not sure, read the appropriate sections before making your selection.

☐ Life sciences ☐ Behavioural & social sciences ☒ Ecological, evolutionary & environmental sciences

For a reference copy of the document with all sections, see nature.com/documents/nr-reporting-summary-flat.pdf

Ecological, evolutionary & environmental sciences study design

All studies must disclose on these points even when the disclosure is negative.

Study description	We calculated extinction debt and colonization credit of 135 vascular plant species of the European Alps by comparing species distribution models with re-survey data of 1576 historical plots, spread over almost 3000 elevational meters. Mismatches between model projections and field observations were interpreted as disequilibria between species distributions and current climatic conditions. We corrected all model projections by a complex calculation of model-based error rates to reduce the potential bias to a minimum.
Research sample	Data are derived from 1576 re-surveyed historical vegetation plots, and comprise in total 1,070 vascular plant taxa of the European Alps.
Sampling strategy	This description of sampling strategy was adapted from Rumpf et al. 2018: Range dynamics of mountain plants decrease with elevation. PNAS 115(8):1848-1853.<br>Following an intensive literature research, we digitized 3,507 historical relevés (i.e., plot data with complete lists of vascular plant species and their cover-abundance values, hereinafter referred to as "plots") of nonforest vegetation in the European Alps. We only included plots that were recorded before 1970, since temperature anomalies accelerated after that time. The primary data sources usually provide a description of the locality, including elevation (meters a.s.l.), slope inclination (degrees), aspect (16 compass directions), plot size (square meters), total vascular plant cover (percent), plant community type, bedrock type, and survey date. If the survey year of a plot was not mentioned, we estimated it as publication year minus 2. Since historical plots had no geographical coordinates, we applied a standardized methodology to relocate them based on this metadata: We used the description of the locality to delineate a polygon within which the plot was situated (e.g., a particular mountain). This polygon was intersected with a digital elevation model with a resolution of 25 × 25 m to delimit those areas matching the topographic specifications of the plot (tolerance ranges: elevation ± 50 m, slope ± 20°, aspect ± 40°). Bedrock information from the original publications was reclassified as calcareous, siliceous, or intermediate. If no information was given, we used the phytosociological classification of the plot in the original publication to assign it to one of these three classes. Subsequently, we further restricted the topographically matching areas by overlay with a substrate layer of the European Alps. From the remaining candidate areas, we selected those within 200 m of trails because authors of the original publications reported using trails to reach their study sites. The network of trails was digitized using aerial images provided by Google Earth. Finally, we defined the coordinates for the resurvey as the centroid of the largest contiguous area remaining after consecutive application of all of these criteria. In cases where no trail was within 200 m of any candidate area, we selected the centroid of the largest contiguous area irrespective of trail distance. Since not all digitized historical plots had full topographic specifications, this sampling design resulted in the relocation of 2011 historical plots. In the vegetation periods of 2014 and 2015, we resurveyed 1,516 of these relocated plots. The Swiss National Park provided another 60 resurveyed historical plots, yielding, in total, 1,576 plots. The resolution of the digital elevation model (25 × 25 m) is coarser than the maximum size of the historical plots (100 m2). Precise plot relocation within the cells was selected based on available additional topographic information (e.g., ridge situation) and coarse vegetation type (rock/scree, snow bed, meadow/heath, tall herbs, bog/swamp). Furthermore, we took care to localize resurvey plots in ± homogeneous vegetation (i.e., avoiding obvious vegetation boundaries) because the intention of historical sampling was to document distinct plant community types.
Data collection	All species within re-survey plots were identified to the most precise taxonomic level possible (i.e., subspecies where applicable). Vouchers were taken to the laboratory if a species could not be identified in the field. Species cover-abundance values were estimated using the scale of Braun–Blanquet (levels +, 1, 2, 3, 4, and 5), and total vascular plant cover per plot was estimated in percent. Re-survey data were recorded by Sabine Rumpf, Martin Schütz, Karl Hülber, Wolfgang Willner, Stefan Dullinger, Christian Kuehs, Siegrun Ertl, Agnes Dellinger, Michaela Sonnleitner, Norbert Helm, Simon Stifter, Erich Kucs, Norbert Sauberer, and Christian Gilli. Terminal falling velocity of plant seeds was recorded using the velocimeter of the Goethe University Frankfurt, Germany, by Sabine Rumpf and Gerald Gimpl. Length, height, width and weight of plant seeds was measured using a calibrated microscope and a stone table balance by Marina Stehlik and Gerald Gimpl.
Timing and spatial scale	Fieldwork was carried out in the vegetation periods of 2014 and 2015 on a daily basis as long as weather conditions permitted it. In 2014 fieldwork lasted from 03/07 – 11/09, and from 24/06 – 11/09 in the year 2015. Re-surveyed plots are located in the European Alps (Austria, Switzerland, Germany, Italy, Slovenia), please see https://phaidra.univie.ac.at/view/o:630655 for coordinates.
Data exclusions	We excluded i) species from the genera Alchemilla and Taraxacum from analyses, because these apomictic taxa have undergone extensive taxonomic revisions; ii) all tree species, because they were only represented by seedlings or saplings in our re-survey; iii) all species that had less than 40 occurrences in the historical data set to achieve sufficiently informed species distribution models; and iv) all species whose historical lower range limit was not covered by our data set since model parametrization is likely unreliable for these species.
Reproducibility	We did not attempt to reproduce our field data. However, all observers working in the re-survey campaign had to self-evaluate all species with respect to the certainty with which they could identify them in the field. Species that were recorded "uncertainly" by any observer without a voucher for postdetermination taken were combined with those with which they are potentially confounded

in aggregates.

Randomization	As the geographic position of re-surveyed plots did not exactly match the position of the historical plots, we used a procedure similar to a k-fold cross-validation to standardize error probabilities when projecting to both historical and recent data. For each species, the data set was randomly split into ten parts, each containing 10% of the plots with equal distributions of presences and absences as in the entire data set. For each of these ten parts, a model was then calibrated with the left out 90% of the plots and projections were made onto the focal 10%. This procedure was hence repeated 10 times to produce projections of the species' presence or absence at each plot under both the current and historical climatic conditions. To avoid possible bias from splitting the data randomly into ten parts, we repeated this entire process ten times, resulting in ten presence/absence projections per plot (pair) and species. We then used a simple majority rule on the subset of these ten projections where the underlying model had reached a True Skill Statistic score > 0.6 to distinguish final presence and absence projections per plot, and determined it as presence in case of a tie.
Blinding	Recent re-surveys were conducted blind, i.e. botanists working in the field did not receive historical species lists.

Did the study involve field work? ☒ Yes ☐ No

Field work, collection and transport

Field conditions	Fieldwork was conducted during the vegetation periods of 2014 and 2015 in 282 man days. Climatic conditions such as temperature and rainfall varied on a day to day basis.
Location	Coordinates, elevation, inclination and exposition of each re-surveyed plot can be accessed online in the Phaidra database at https://phaidra.univie.ac.at/view/o:630655
Access and import/export	Permits were obtained for fieldwork in national parks. For the national park Hohe Tauern, Austria, a permit was obtained from Christina Pertl from the Bezirkshauptmannschaft Spittal an der Drau on 23.06.2015, number 31/3/15. For the Swiss national park, a permit was obtained from Ruedi Haller from the Swiss national park on 28.04.2015, number CH-5369. For the national park Berchtesgaden, Germany, a permit was obtained from Sigrid Rossiwal from the Regierung von Oberbayern on 15.04.2015, number 55.1-8645-3-2015. Due to unknown reasons, it was withdrawn by Doris Huber from the national park Berchtesgaden after 2 days in the field and all further fieldwork in the area was aborted.
Disturbance	Samples of species were only taken if a species could not be identified safely in the field.

Reporting for specific materials, systems and methods

We require information from authors about some types of materials, experimental systems and methods used in many studies. Here, indicate whether each material, system or method listed is relevant to your study. If you are not sure if a list item applies to your research, read the appropriate section before selecting a response.

Materials & experimental systems

n/a	Involved in the study	
☒	☐	Antibodies
☒	☐	Eukaryotic cell lines
☒	☐	Palaeontology
☐	☒	Animals and other organisms
☒	☐	Human research participants
☒	☐	Clinical data

Methods

n/a	Involved in the study	
☒	☐	ChIP-seq
☒	☐	Flow cytometry
☒	☐	MRI-based neuroimaging

Animals and other organisms

Policy information about studies involving animals; ARRIVE guidelines recommended for reporting animal research

Laboratory animals	none
Wild animals	none
Field-collected samples	Samples collected in the field were herbarized during field work.
Ethics oversight	No ethical approval was required. No experiments on living organisms were conducted.

Note that full information on the approval of the study protocol must also be provided in the manuscript.
